# In Vitro Mutagenic and Genotoxic Assessment of Anatoxin-a Alone and in Combination with Cylindrospermopsin

**DOI:** 10.3390/toxins15070458

**Published:** 2023-07-13

**Authors:** Cristina Plata-Calzado, Leticia Diez-Quijada, Concepción Medrano-Padial, Ana I. Prieto, Ana M. Cameán, Angeles Jos

**Affiliations:** Area of Toxicology, Faculty of Pharmacy, Universidad de Sevilla, Profesor García González 2, 41012 Seville, Spain; cpcalzado@us.es (C.P.-C.); ldiezquijada@us.es (L.D.-Q.); cmpadial@us.es (C.M.-P.); camean@us.es (A.M.C.);

**Keywords:** genotoxicity, mutagenicity, anatoxin-a, cylindrospermopsin

## Abstract

Anatoxin-a (ATX-a) is a cyanobacterial toxin whose occurrence has been reported worldwide and has attracted increasing scientific interest due to its toxicity. Moreover, in nature, ATX-a usually appears together with other cyanotoxins, such as cylindrospermopsin (CYN), so possible interaction phenomena could happen and should be considered for risk assessment purposes. For this reason, the aim of this work was to explore the potential mutagenicity and genotoxicity of pure ATX-a and an ATX-a/CYN mixture using a battery of in vitro assays, including the bacterial reverse-mutation assay in *Salmonella typhimurium* (OECD 471) and the micronucleus test (MN) (OECD 487) on L5178Y Tk^+/−^ cells. The results showed that ATX-a was not mutagenic either alone or in combination with CYN under the conditions tested. Nevertheless, genotoxic effects were observed for both ATX-a and its mixture with CYN following the in vitro MN assay. The genotoxicity exhibited by ATX-a was only observed in the absence of S9 mix, whereas in the cyanotoxin mixture the concentration-dependent genotoxicity of ATX-a/CYN in vitro was observed only in the presence of S9. Thus, the toxicity induced by cyanotoxin mixtures may vary from that produced by toxins alone, and consequently more studies are necessary in order to perform more realistic risk assessments.

## 1. Introduction

Anthropogenic activities and climate change are increasing the frequency and extension of cyanobacterial blooms capable of producing cyanotoxins that can impact ecosystems and human health [1,2]. The primary route of exposure to cyanotoxins for humans is through the intake of contaminated water and food, such as crops, fish, vegetables, and algae-based supplements [3,4,5,6,7]. Anatoxin-a (ATX-a) is a cyanobacterial toxin that has been reported worldwide and has attracted increasing scientific interest due to its toxicity [8,9]. This cyanotoxin is a secondary amine alkaloid produced by numerous genders of cyanobacteria such as *Anabaena*, *Aphanizomenon*, *Oscillatoria,* and *Microcystis* [10]. Numerous animal poisonings have been described after exposure to ATX-a, producing symptoms such as tremors, paresthesia, convulsions, muscle paralysis, and hypoxia [8,11].

Moreover, different studies have shown that the nervous system is the main target of ATX-a activity. Thus, in its mechanism of action, this cyanotoxin is a nicotinic agonist that competes with acetylcholine for binding to neuronal nicotinic acetylcholine receptors [12,13]. ATX-a binding produces a neuromuscular blockade that can lead to death by respiratory arrest in acute intoxications [14]. However, additional effects attributed to ATX-a include cytotoxic effects [15,16], immunotoxicity [17,18], effects on development and reproduction [19,20,21], and heart effects [22,23].

Nevertheless, there are almost no studies related to ATX-a’s genotoxicity and mutagenicity. Thus, Sieroslawska [24] assessed the mutagenic potential of cyanobacterial extracts and pure ATX-a using the Ames test. The results showed mutagenic activity in *Salmonella typhimurium* strains TA98 and TA100 after exposure to cyanobacterial extracts that contained ATX-a, whereas pure ATX-a was not mutagenic. Another study conducted in *S. Typhimurium* TA1535 showed the genotoxicity of ATX-a without the S9 fraction using the UmuC Easy CS assay [25]. In contrast, these same authors did not observe DNA damage on carp leukocytes when exposed to 0.5 µg/mL ATX-a in the comet assay [26]. These contradictory results about ATX-a’s genotoxicity make necessary further studies to elucidate its genotoxic potential. Moreover, the genotoxic potential of ATX-a has not yet been explored using the micronucleus (MN) test, an assay recommended by the European Food Safety Authority (EFSA) for the genotoxicity evaluation of food contaminants [27].

On the other hand, it should also be noted that in nature there are often multiple toxins in cyanobacterial blooms, so interactions between cyanotoxins may occur [5]. In this sense, the simultaneous occurrence of ATX-a and cylindrospermopsin (CYN) has been reported in aquatic environments [28,29,30]. Moreover, it has been observed that the toxic effects of this toxin are usually more potent in combination with other cyanotoxins such as CYN or MC-LR compared to the individual compound [18,31].

CYN is a tricyclic guanidine alkaloid, which mainly affects the liver, although it also damages other organs such as the kidneys, heart, lungs, spleen, thymus, or nervous system [32]. Several mechanisms have been associated with CYN toxicity, notably the irreversible inhibition of protein synthesis [33,34], the induction of oxidative stress [35,36,37], and reductions in glutathione (GSH) levels [38]. Furthermore, the previous studies available in the scientific literature described CYN’s genotoxicity [39,40,41].

Specifically, previous studies performed by our research group revealed in vitro genotoxic effects produced by CYN alone when the battery of tests recommended by the EFSA (an Ames test and micronucleus test) was applied [40]. In addition, these effects have also been reported for the CYN/MC-LR mixture (1:10) under the same laboratory conditions [40,42]. However, lower concentrations of CYN alone compared to the cyanotoxin mixture are necessary to observe these effects, showing differences in the toxic response.

Despite frequent confluences of cyanotoxins occurring in nature, studies focusing on the effects of CYN in combination with other cyanotoxins are scarce. In this sense, due to the different chemical structure and mechanisms of action of ATX-a and CYN and their simultaneous occurrence in nature, possible interaction phenomena can occur, and this should be considered in the risk assessment of these cyanotoxins [7]. Furthermore, the EFSA has emphasized the necessity for additional studies focusing on the toxicity of cyanotoxin mixtures [5], with the exploration of the genotoxic potential of the ATX-a/CYN mixture being of great interest due to its possible presence in water and food.

Thus, the purpose of this work was to explore the potential mutagenicity and genotoxicity of pure ATX-a and an ATX-a/CYN mixture using the basic battery of in vitro assays recommended by the EFSA, including the bacterial reverse-mutation assay in *Salmonella typhimurium* (the Ames test, OECD 471 [43]) to detect gene mutations and the MN test (OECD 487 [44]) on L5178Y Tk^+/−^ cells to detect structural and numerical chromosome aberrations. All assays were conducted both with and without the presence of the S9 mix to evaluate the effect of metabolic activation on the genotoxicity of these toxins.

## 2. Results

### 2.1. Ames Test

ATX-a produced no changes in *S. typhimurium* strains TA98, TA100, TA1535, and TA1537 without metabolic activation (Table 1). However, the number of revertant colonies per plate was significantly increased in the TA102 strain compared to the control group for the same experimental conditions. In the presence of metabolic activation system S9 (S9 mix), significant changes were only observed in strains TA100 and TA102, both showing a reduction in the number of revertant colonies (at 1 µ/mL and 0.125–0.25 µg/mL, respectively).

In relation to the cyanotoxin mixture, ATX-a/CYN only produced changes in strain TA102 in the absence of the S9 mix, showing a decrease in bacterial growth at the exposure concentration of 1 µg/mL (Table 2). However, in the presence of the metabolic fraction S9, the effect was the opposite, as a significant increase in the number of colonies was observed in the TA 102 and TA 1535 strains (at 0.5 µg/mL and 0.125 µg/mL, respectively).

None of the strains showed a mutagenicity index (MI) higher than 2, so neither ATX-a nor its mixture with CYN showed mutagenic potential under the conditions tested.

Furthermore, the detected changes in bacterial growth did not follow a concentration–response pattern in any of the tested strains.

In both cases, a significant increase in revertant colonies and a MI > 2 were observed after exposure of the *S. typhimurium* strains to the positive controls, confirming the sensitivity of the present experiment. Moreover, DMSO (solvent control) did not produce statistically significant changes compared to the negative controls.

### 2.2. Micronucleus Test

In the absence of the S9 fraction, significant increases in the percentage of BNMN were observed at the higher tested concentrations of ATX-a (10 and 20 µg/mL) with both short (4 h) and long (24 h) exposure periods (Table 3). Furthermore, this observed increase in MN showed a concentration-dependent trend. Nevertheless, no significant change in the BNMN parameter was noted in the presence of S9 mix after 4 h of exposure to the same range of ATX-a concentrations (from 1.25 to 20 µg/mL).

When the ATX-a/CYN mixture was tested in the absence of S9 mix, only an increase in the percentage of BNMN was detected after a short exposure period (4 h) at the concentration of 1.35 µg/mL (Table 4). However, no significant increase in this parameter was observed after longer periods (24 h). In addition, after 24 h of exposure to the highest concentration assessed, MN could not be quantified due to a cell viability decrease. In contrast, in the presence of S9 mix, the frequency of BNMN was significantly increased from 0.5 µg/mL ATX-a/CYN, with the maximum value being found at the highest concentration assayed (µg/mL).

All positive controls used in the assay, both for clastogenic and aneugenic substances, showed a significant increase in the BNMN percentage compared to their negative control group, indicating the validity of the assays performed.

In addition, after the cell count, all CBPI values remained within the range indicated by the OECD for adequate performance of the assay.

## 3. Discussion

Exposure to cyanotoxins poses a potential risk to public health. To address this concern, the EFSA has emphasized the importance of a better understanding of the toxicological properties of cyanotoxins [5]. Regarding ATX-a, the literature data confirm its acute neurotoxic effects [13,45,46]; nevertheless, there is almost no information on the genotoxicity of this alkaloid [41].

In the bacterial reverse mutation test, ATX-a showed no mutagenic response in any of the *S. typhimurium* strains tested under the conditions of the study. There are nearly no data on the possible mutagenic properties of ATX-a. Only one report was found using the Ames test, which tested a lower toxin concentration range (0.312–10 µg/mL), also obtaining negative results [24]. Controversially, the same authors detected genotoxic effects only in the strain 1535 after the exposure of ATX-a in the absence of S9 when performing the UmuC Easy CS assay [25]. Although both the Ames and UmuC assays are carried out with *Salmonella tyhphimurum* strains, the endpoints measured in those tests differ, hindering a comparison between the results obtained. Moreover, while the MI was lower than 2 in all cases, some variations in the number of revertant colonies were found in strains related with base-pair substitutions (TA100 and TA102) [47,48]. Specifically, in the absence of metabolic fraction S9, an increase was observed at almost all concentrations tested in TA102, a strain that detects oxidative DNA damage [49]. This finding could be mainly explained by previous studies that demonstrated that ATX-a can cause an increase in ROS levels parallel to the dose and time, causing DNA fragmentation and alterations in oxidant parameters [9,15,41,50,51].

In addition, the assessment of the toxicological profile of cyanotoxin mixtures is of great importance given the high probability of exposure to multiple toxins in nature, as stated by the EFSA [5]. In this regard, other authors have considered the study of the genotoxic potential of another toxin mixture, namely CYN/MC-LR [42,52]. In our case, the genotoxicity of the ATX-a/CYN combination has been evaluated. In the Ames test, our results showed an MI index of less than 2 for all concentrations of cyanotoxin mixture and strains assayed. Considering that previous studies have shown that CYN alone is not mutagenic in a similar range of concentrations (from 0.625–10 µg/mL) [24,40], together with the results obtained from ATX-a alone in the present study, these results suggest that the ATX-a/CYN combination does not change the mutagenic responses of individual toxins. This is in line with other studies that assessed the potential mutagenicity of cyanotoxin mixtures, where no mutagenicity was observed in the Ames test after exposure to the CYN/MC-LR mixture at a 1:10 ratio [42] or the equimolar mixture of CYN/MC-LR/ATX-a at 1 µg/mL [24]. Our findings seem to indicate that ATX-a alone or in combination with CYN does not produce mutagenicity, even at concentrations higher than those typically found in the environment.

It is a known fact that a single toxicity assay cannot detect all toxic mechanisms caused by any toxin. For this reason, in addition to assessing mutagenicity, an evaluation of the genotoxicity of ATX-a was carried out in this work. In this respect, the in vitro MN assay was employed because it can detect both structural and numerical chromosomal damages. Our results showed significant increases in BNMN in the L5178YTk^+/−^ cell line after 4 h and 24 h of exposure to the high ATX-a concentrations (10 and 20 µg/mL) tested in the absence of the S9 mix. To our knowledge, no other in vitro MN test has been performed with pure ATX-a. In contrast, Abramsson-Zetterberg et al. [53] observed no genotoxic effects in an MN assay in human lymphocytes in the absence of S9 following exposure to a cyanotoxin extract (0.25–2 mg/mL) suspected to contain ATX-a. Nevertheless, these authors did not conclude whether the neurotoxin was present in this extract. Another explanation for this discrepancy in the results could be the possible interactions between the toxins present in the extract tested by Abramsson-Zetterberg et al. [53].

On the other hand, in the presence of S9 mix, no significant increase in %BNMN was observed in the same concentration range of ATX-a (0.125–20 µg/mL). These results suggest that ATX-a metabolites exert less genotoxic effects than the parent compound. There are no data regarding ATX-a’s metabolism [54], although it is known that metabolism can both reduce or increase the toxicity of cyanotoxins, as is the case of microcystins [55] and CYN [56], respectively.

As for the ATX-a/CYN combination, an increase in micronuclei was observed mainly with the S9 fraction. Taking into account that ATX-a in the presence of the S9 fraction did not produce an increase in %BNMN, the potential genotoxic damage could be due to CYN metabolites present in the mixture. In this sense, it has been reported that the metabolic transformation of CYN is crucial for its toxin-induced toxicity, and particularly for its genotoxicity [57,58]. However, CYN metabolites have not been identified so far [40]. Specifically, the need for CYN’s metabolic activation by cytochrome P-450 enzymes for its genotoxic activity has been reported [56].

In relation to the increase observed at 1.35 µg/mL in ATX-a/CYN in the absence of the S9 fraction, Puerto et al. [40] showed no genotoxic effects of CYN in the absence of S9 mix, whereas in our study ATX-a alone produced increases in BNMN at higher concentrations (10 and 20 µg/mL). These results suggest an enhancement of the genotoxic response of CYN when it is combined with ATX-a. In a similar way, it has been reported that MC-LR combined with aflatoxin B1 (AFB1) presented a significant increase in micronuclei as well as increased DNA damage in the comet assay compared to the damage induced by a single exposure of MC-LR and AFB1 [59]. Hercog et al. [52], in contrast, showed an antagonistic interaction of the MC-LR/CYN combination with lower levels of DNA breaks in the mixture compared to single toxins. Although it is difficult to underline the toxic mechanism of action of the ATX-a/CYN combination, this finding is a first step to bridging the existing gap in hazard identification and characterization regarding the possible additive, synergistic, or antagonistic effects of different cyanotoxins described by the EFSA [5].

Moreover, it is noteworthy that the highest concentration of the ATX-a/CYN mixture evaluated (1.35 µg/mL) in the absence of S9 could not be quantified due to low cell viability after 24 h of exposure. Previous studies have already shown a decrease in the nuclear division index at this concentration of CYN [40,42], so the combination with ATX-a could enhance this situation after long periods of exposure. This is in agreement with Takser et al. [18], who reported an increase in cell death with the CYN/MC-LR/ATX-a mixture after 72 h of exposure compared to a single CYN exposure.

In the presence of S9, CYN alone showed a genotoxic response from low concentrations (0.25 µg/mL) [40], whereas in this study the mixture with ATX-a showed this effect at 0.5 µg/mL. This finding suggests that ATX-a reduces, in this case, the CYN response.

To the best of our knowledge, there are no other studies that have explored the genotoxic potential of the ATX-a/CYN mixture using an in vitro MN assay. However, other studies have also shown a different response following exposure to individual cyanotoxins or cyanotoxin mixtures. Thus, the CYN/MC-LR mixture also reduced the genotoxic response of the CYN individual treatment in mouse lymphoma cells [42] in the MN test. This variety of results highlights the importance of the characterization of preparations containing a mixture of toxins, since they may have antagonistic, potentiating, or synergistic effects as compared to other toxins tested individually.

Overall, it is challenging to make a conclusive statement about the genotoxicity of ATX-a and its combination with CYN due the scarcity of relevant studies in the scientific literature. However, this study marks the first comprehensive investigation, utilizing the genotoxicity testing battery recommended by EFSA for ATX-a and its mixture with CYN. The findings indicate that the mixture commonly found in nature exhibits greater genotoxicity compared to ATX-a alone.

## 4. Conclusions

ATX-a showed no mutagenic effects either alone or in combination with CYN under the conditions tested in the Ames test. Nevertheless, genotoxic effects were observed for both ATX-a and its mixture with CYN following the in vitro MN assay in the cell line L5178Y Tk^+/−^. Furthermore, the genotoxicity exhibited by ATX-a was only observed in the absence of the S9 mix, whereas in the ATX-a/CYN cyanotoxin mixture, concentration-dependent genotoxicity in vitro was observed only in the presence of S9. It is important to evaluate the toxic effects produced by cyanotoxin mixtures, as these may vary from those produced by the toxins alone, in order to make a more realistic risk assessment.

## 5. Materials and Methods

### 5.1. Chemicals

The (±) anatoxin-a fumarate (purity > 98.0%) and cylindrospermopsin (purity 95%) standards were provided by Enzo Life Sciences (Lausen, Switzerland). All chemical reagents were obtained from Gibco (Biomol, Sevilla, Spain), Sigma–Aldrich (Madrid, Spain), Moltox (Trinova, Biochem, Germany), and C-Viral S.L. (Sevilla, Spain).

### 5.2. Cells and Culture Conditions

For the MN assay, L5178Y Tk^+/−^ mouse lymphoma cells were used. The cells were cultured and maintained in RPMI 1640 medium supplemented with 10% horse serum, 1% L-glutamine, and 1% penicillin–streptomycin in an incubator with 95% relative humidity and 5% CO_2_ at 37 °C.

Moreover, different histidine auxotrophic strains of *Salmonella typhimurium* (TA98, TA100, TA102, TA1535, and TA1537) were used to perform the Ames test.

### 5.3. Test Solutions

Stock solutions of ATX-a (4000 µg/mL) and CYN (1000 µg/mL) were prepared in milliQ sterile water and stored at −20 °C. Sterile milliQ water for the Ames test or RPMI 1640 medium for the MN assay was used to prepare the exposure concentration solutions. The ATX-a/CYN mixtures were prepared in the same ratio because the concentrations of both cyanotoxins in nature are normally similar [1,60,61].

### 5.4. Ames Test

The bacterial reverse mutation test was performed according to the principles of OECD guideline 471 [43]. The cultures of five *Salmonella typhimurium* histidine-auxotrophic strains (TA98, TA100, TA102, TA1535, and TA1537) were purchased from TRINOVA BIOCHEM GmbH (Germany) and grown according to the provider’s instructions. The mutagenic activity of ATX-a and the ATX-a/CYN mixture was assessed using three technical replicates per concentration with and without the rat liver S9 fraction as the metabolic activation system. Different concentrations of ATX-a (range of 0.125–20 µg/mL) and the ATX-a/CYN mixture (range of 0.125–2 µg/mL) were selected in accordance with Sieroslawska [24] and Puerto et al. [40]. Considering the concentrations of cyanotoxins found in nature [11,62,63], higher concentrations were not analyzed because they were not deemed relevant. In addition, distilled sterile water was used as the negative control and DMSO as the solvent control, along with different positive controls that depended both on the strain and presence or absence of the S9 fraction. (Positive controls without the S9 fraction for TA98: 2-nitrofluorene (20 µg/plate); TA100 and TA1535: azide Na (1.5 μg/plate); TA102: dexon (50 µg/plate); TA1537: 9- aminoacridine (50 µg/plate). Positive controls with S9 fraction for TA98 and TA100: 2-aminofluorene (20 μg/plate); TA102, TA1535, and TA1537: 2-aminoanthracene (10 µg/plate).) The data are given as the number of revertant colonies and mutagenicity indexes (MI).

### 5.5. Micronucleus Test

This test was conducted in accordance with OECD guideline 487 [44]. To this end, L5178Y Tk^+/−^ cells (at 2.0 × 105 cell/mL) were treated with five growing concentrations of ATX-a and the ATX-a/CYN mixture in the absence and presence of S9. The exposure times were 4 h and 24 h in the absence of the S9 fraction and 4 h in the presence of the metabolic fraction.

The test concentrations were selected based on the previous cytotoxicity results obtained with ATX-a and the CYN/ATX-a mixture according to OECD guideline 487 [40,42]. Thus, these tests showed no cytotoxicity for ATX-a alone, so the concentration range was 1.125–20 µg/mL. In contrast, the CYN/ATX-a mixture showed different cytotoxicity levels in the absence and presence of S9, which limited the range of concentrations to be tested to 0.084–1.35 µg/mL (without the S9 fraction for 4 and 24 h) and 0.125–2 µg/mL (with the S9 fraction for 4 h). In addition, as the proportions in which both toxins appear in nature are similar, we decided to evaluate the mixture at a ratio 1:1.

A negative control (RPMI medium) and positive controls (0.0125 µg/mL colchicine and 0.0625 µg/mL mitomycin without S9 mix and 8 µg/mL cyclophosphamide in the presence of the S9 fraction) were used. The cells were exposed for 4 h or 24 h, after which they were exposed to cytochalasin B (6 µg/mL) for 20 h. Then, the cultures were subjected to a hypotonic shock with 0.051 M KCl and fixed with Carnoy solution. Subsequently, the cell suspension was placed onto the microscope slides and stained with 10% Giemsa. According to the recommendations of OECD 487 (2016), the frequency rates of binucleated cells with micronuclei (BNMN) were scored in at least 2000 binucleated cells per concentration and the cytokinesis block proliferation index (CBPI) scores were analyzed for at least 1000 cells per concentration.

### 5.6. Statistical Analysis

A statistical analysis was conducted using an analysis of variance (ANOVA) followed by Dunnett’s multiple comparisons test to compare the exposed samples with the negative control. The Kolmogorov–Smirnov test was used to check the distribution of the results. All data were analyzed with Graph-Pad Prism 8.0.1 (Graph-Pad Prism 8 Software Inc., La Jolla, CA, USA). The results are expressed as means ± standard deviations (SDs). Differences were considered significant at * p < 0.05, ** p < 0.01, *** p < 0.001, and **** p < 0.0001.

## Figures and Tables

**Table 1:** Effect of ATX-a on the Ames test performed in triplicate. Milli Q water (100 µL) was used as the negative control and DMSO (10 μL) as the solvent for positive controls. Positive controls without the S9 fraction for TA98: 2-nitrofluorene (20 µg/plate); TA100 and TA1535: azide Na (1.5 μg/plate); TA102: dexon (50 µg/plate); TA1537: 9-aminoacridine (50 µg/plate). Positive control with the S9 fraction for TA98 and TA100: 2-aminofluorene (20 μg/plate); TA102, TA1535, and TA1537: 2-aminoanthracene (10 µg/plate). The values are expressed as means ± SDs for revertants/plate and the mutagenicity index (MI). Note: * p < 0.05, ** p < 0.01, and **** p < 0.0001, significantly different from negative controls.

Concentration (µg/mL)		TA98				TA100				TA102				TA1535				TA1537			
		−S9	MI	+S9	MI	−S9	MI	+S9	MI	−S9	MI	+S9	MI	−S9	MI	+S9	MI	−S9	MI	+S9	MI
ATX-a	Negative control	24 ± 6	-	29 ± 4	-	86 ± 13	-	96 ± 5	-	179 ± 49	-	269 ± 48	-	15 ± 3	-	14 ± 1	-	13 ± 3	-	12 ± 2	-
	0.125	23 ± 7	1.0	31 ± 3	1.1	75 ± 16	0.9	84 ± 5	0.9	220 ± 17 *	1.2	214 ± 36 **	0.8	16 ± 4	1.0	10 ± 3	0.7	12 ± 6	0.9	12 ± 3	1.1
	0.25	14 ± 4	0.6	29 ± 3	1.0	85 ± 3	1.0	88 ± 12	0.9	180 ± 63	1.0	212 ± 12 **	0.8	16 ± 2	1.0	13 ± 2	1.0	11 ± 1	0.8	10 ± 4	0.9
	0.5	25 ± 6	1.1	32 ± 2	1.1	86 ± 7	1.0	79 ± 12	0.8	313 ± 18 ****	1.8	233 ± 31	0.9	16 ± 4	1.0	13 ± 4	1.0	7 ± 1	0.5	14 ± 2	1.2
	1	28 ± 3	1.2	30 ± 6	1.0	83 ± 3	1.0	72 ± 15 **	0.8	255 ± 8 ****	1.4	231 ± 27	0.9	23 ± 4	1.5	13 ± 3	1.0	8 ± 2	0.6	13 ± 1	1.1
	2	25 ± 11	1.1	30 ± 8	1.0	74 ± 15	0.9	91 ± 16	0.9	292 ± 38 ****	1.6	255 ± 43	0.9	9 ± 5	0.6	15 ± 5	1.1	11 ± 1	0.8	17 ± 4	1.5
	4	26 ± 4	1.1	32 ± 9	1.1	87 ± 20	1.0	88 ± 8	0.9	265 ± 23 ****	1.5	280 ± 20	1.0	19 ± 2	1.3	10 ± 2	0.7	13 ± 2	1.0	8 ± 5	0.7
	20	26 ± 6	1.1	19 ± 7	0.7	91 ± 4	1.1	87 ± 14	0.9	251 ± 35 ****	1.4	267 ± 17	1.0	12 ± 4	0.8	15 ± 1	1.1	12 ± 2	0.9	10 ± 2	0.8
	Positive control	712 ± 52 ****	30.1	627 ± 176 ****	21.4	404 ± 20 ****	4.7	420 ± 35 ****	4.4	599 ± 23 ****	3.4	711 ± 35 ****	2.6	991 ± 183 ****	64.6	173 ± 45 ****	12.7	179 ± 126 ****	13.7	149 ± 14 ****	12.8
DMSO		18 ± 6	0.7	37 ± 4	1.3	86 ± 13	1.0	90 ± 11	0.9	255 ± 20 ****	1.4	176 ± 62 ****	0.7	22 ± 5	1.4	15 ± 5	1.1	8 ± 4	0.6	7 ± 4	0.6

**Table 2:** Effect of ATX-a/CYN mixture on the Ames test performed in triplicate. Milli Q water (100 µL) was used as the negative control and DMSO (10 μL) as the solvent for positive controls. Positive controls without the S9 fraction for TA98: 2-nitrofluorene (20 µg/plate); TA100 and TA1535: azide Na (1.5 μg/plate); TA102: dexon (50 µg/plate); TA1537: 9-aminoacridine (50 µg/plate). Positive control with the S9 fraction for TA98 and TA100: 2-aminofluorene (20 μg/plate); TA102, TA1535, and TA1537: 2-aminoanthracene (10 µg/plate). The values are given as means ± SDs for revertants/plate and the mutagenicity index (MI). Note: * p < 0.05, ** p < 0.01, and **** p < 0.0001, significantly different from negative controls.

Concentration (µg/mL)		TA98				TA100				TA102				TA1535				TA1537			
		−S9	MI	+S9	MI	−S9	MI	+S9	MI	−S9	MI	+S9	MI	−S9	MI	+S9	MI	−S9	MI	+S9	MI
ATX-a + CYN	Negative control	20 ± 3	-	25 ± 2	-	56 ± 12	-	46 ± 4	-	261 ± 44	-	383 ± 41	-	11 ± 3	-	9 ± 5	-	10 ± 2	-	12 ± 4	-
	0.125	21 ± 7	1.0	30 ± 14	1.2	62 ± 9	1.1	55 ± 11	1.2	230 ± 21	0.9	433 ± 8	1.1	6 ± 4	0.5	16 ± 4 *	1.7	9 ± 3	1.0	11 ± 4	1.0
	0.25	18 ± 2	0.9	21 ± 9	0.9	58 ± 6	1.0	44 ± 3	0.9	279 ± 15	1.1	321 ± 23	0.8	12 ± 6	1.2	11 ± 2	1.2	8 ± 2	0.8	11 ± 6	0.9
	0.5	37 ± 3	1.8	20 ± 3	0.8	47 ± 11	0.8	56 ± 17	1.2	265 ± 38	1.0	519 ± 106 **	1.4	13 ± 6	1.2	12 ± 3	1.3	9 ± 2	1.0	11 ± 3	0.9
	1	12 ± 3	0.6	19 ± 5	0.8	56 ± 12	1.0	50 ± 4	1.1	146 ± 24 ****	0.6	339 ± 6	0.9	13 ± 4	1.2	11 ± 4	1.2	7 ± 3	0.8	13 ± 4	1.1
	2	16 ± 3	0.8	15 ± 7	0.6	53 ± 3	1.0	56 ± 10	1.2	277 ± 58	1.1	395 ± 61	1.0	13 ± 3	1.3	9 ± 2	0.9	5 ± 3	0.5	9 ± 4	0.8
	Positive control	555 ± 55 ****	27.3	1803 ± 751 ****	73.1	493 ± 94 ****	8.9	459 ± 88 ****	9.9	629 ± 20 ****	2.4	897 ± 176 ****	2.3	425 ± 20 ****	39.9	49 ± 12 ****	5.2	225 ± 55 ****	23.3	134 ± 17 ****	11.5
DMSO		19 ± 3	1.0	20 ± 3	0.8	49 ± 10	0.9	44 ± 9	0.9	272 ± 25	1.0	459 ± 24	1.2	8 ± 3	0.7	9 ± 3	1.0	7 ± 1	0.7	10 ± 5	0.8

**Table 3:** Results of the MN test in L5178YTk^+/−^ mouse lymphoma cells exposed to pure ATX-a. The genotoxicity assay was conducted in the absence and presence of the S9 fraction. The data are expressed as means ± SDs. Note: * p < 0.05, ** p < 0.01, **** p < 0.0001 in comparison to negative control group values.

Experimental Group	Absence of S9								Presence of S9			
	Exposure Time (h)	Concentrations (µg/mL)	BNMN (%) ± SD	CBPI ± SD	Exposure Time (h)	Concentrations (µg/mL)	BNMN (%) ± SD	CBPI ± SD	Exposure Time (h)	Concentrations (µg/mL)	BNMN (%) ± SD	CBPI ± SD
Negative control	4	-	1.1 ± 0.3	1.9 ± 0.1	24		0.6 ± 0.2	1.5 ± 0.0	4	-	1.2 ± 0.3	1.7 ± 0.0
Positive control	4	Mitomycin C 0.0625	3.7 ± 0.7 ****	1.9 ± 0.1	24	Mitomycin C 0.0625	4.7 ± 1.1 ****	1.6 ± 0.0	4	Cyclophosphamide 8	4.0 ± 0.6 *	1.7 ± 0.0
		Colchicine 0.0125	4.5 ± 0.9 ****	2.2 ± 0.1 ****		Colchicine 0.0125	3.1 ± 0.4 ****	1.7 ± 0.1 **				
ATX-a	4	1.25	2.1 ± 0.4	1.9 ± 0.1	24	1.25	0.8 ± 0.3	1.5 ± 0.0	4	1.25	1.7 ± 0.2	1.8 ± 0.0
		2.5	2.0 ± 0.3	2.0 ± 0.1 **		2.5	0.8 ± 0.2	1.5 ± 0.0		2.5	0.8 ± 0.2	1.7 ± 0.1
		5	2.3 ± 0.4	1.9 ± 0.1		5	0.9 ± 0.2	1.5 ± 0.0		5	0.6 ± 0.3	1.5 ± 0.1 *
		10	2.4 ± 0.4 *	1.9 ± 0.1		10	1.6 ± 0.4 **	1.5 ± 0.0		10	0.8 ± 0.5	1.6 ± 0.1
		20	2.7 ± 0.4 **	1.9 ± 0.1		20	1.9 ± 0.5 **	1.5 ± 0.0		20	1.2 ± 0.5	1.7 ± 0.1

BNMN (%): MN present per 100 binucleated cells; CBPI: cytokinesis block proliferation index.

**Table 4:** Results of the MN test in L5178YTk^+/−^ mouse lymphoma cells exposed to the ATX-a/CYN mixture (1:1). The genotoxicity assay was conducted in the absence and presence of the S9 fraction. The data are expressed as means ± SDs. Note: * p < 0.05, ** p < 0.01, *** p < 0.001 in comparison to negative control group values.

Experimental Group	Absence of S9								Presence of S9			
	Exposure Time (h)	Concentrations (µg/mL)	BNMN (%) ± SD	CBPI ± SD	Exposure Time (h)	Concentrations (µg/mL)	BNMN (%) ± SD	CBPI ± SD	Exposure Time (h)	Concentrations (µg/mL)	BNMN (%) ± SD	CBPI ± SD
Negative control	4	-	1.4 ± 0.2	1.8 ± 0.1	24	-	0.5 ± 0.6	1.6 ± 0.0	4	-	0.5 ± 0.3	1.6 ± 0.1
Positive control	4	Mitomycin C 0.0625	3.7 ± 0.7 ***	1.8 ± 0.0	24	Mitomycin C 0.0625	1.7 ± 0.4 **	1.6 ± 0.1	4	Cyclophosphamide 8	1.2 ± 0.3 *	1.6 ± 0.0
		Colchicine 0.0125	3.0 ± 0.3 *	1.9 ± 0.0 *		Colchicine 0.0125	1.6 ± 0.7 **	1.8 ± 0.1				
ATX-a/CYN	4	0.084	1.4 ± 0.5	1.8 ± 0.0	24	0.084	0.8 ± 0.5	1.5 ± 0.1	4	0.125	0.6 ± 0.3	1.7 ± 0.1
		0.168	1.4 ± 0.3	1.7 ± 0.0		0.168	0.2 ± 0.2	1.5 ± 0.1		0.25	1.1 ± 0.3	1.6 ± 0.0
		0.337	1.4 ± 0.2	1.7 ± 0.1		0.337	0.6 ± 0.5	1.5 ± 0.1		0.5	1.3 ± 0.6 *	1.6 ± 0.0
		0.675	1.6 ± 0.4	1.7 ± 0.0		0.675	1.3 ± 0.4	1.6 ± 0.0		1	1.2 ± 0.3 *	1.6 ± 0.0
		1.35	2.9 ± 0.7 *	1.8 ± 0.1		1.35	-	-		2	1.6 ± 0.9 **	1.6 ± 0.0

BNMN (%): MN present per 100 binucleated cells; CBPI: cytokinesis block proliferation index.

## Data Availability

Not applicable.
